# Efficacy and Safety of Cangrelor in Women Versus Men During Percutaneous Coronary Intervention: Insights From the Cangrelor versus Standard Therapy to Achieve Optimal Management of Platelet Inhibition (CHAMPION PHOENIX) Trial

**DOI:** 10.1161/CIRCULATIONAHA.115.017300

**Published:** 2016-01-18

**Authors:** Michelle L. O’Donoghue, Deepak L. Bhatt, Gregg W. Stone, Ph. Gabriel Steg, C. Michael Gibson, Christian W. Hamm, Matthew J. Price, Jayne Prats, Tiepu Liu, Efthymios N. Deliargyris, Kenneth W. Mahaffey, Harvey D. White, Robert A. Harrington

**Affiliations:** From Cardiovascular Division, Brigham and Women’s Hospital, Boston, MA (M.L.O., D.L.B.); Columbia University Medical Center and the Cardiovascular Research Foundation, New York, NY (G.W.S.); FACT, DHU FIRE, Université Paris-Diderot, Sorbonne Paris-Cité, France (G.S.); LVTS INSERM U-1148, Hôpital Bichat, HUPNVS, AP-HP, Paris, France (G.S.); NHLI, Imperial College, Royal Brompton Hospital, London, United Kingdom (G.S.); Beth Israel Deaconess Medical Center, Division of Cardiology, Boston, MA (C.M.G.); Kerckhoff Heart and Thorax Center, Bad Nauheim, Germany (C.W.H.); Scripps Clinic and Scripps Translational Science Institute, La Jolla, CA (M.J.P.); The Medicines Company, Parsippany, NJ (J.P., T.L., E.N.D.); Stanford University Medical School, Stanford, CA (K.W.M., R.A.H.); and Green Lane Cardiovascular Service, Auckland, New Zealand (H.D.W.).

**Keywords:** ADP receptor antagonist, cangrelor, clopidogrel, percutaneous coronary intervention

## Abstract

**Background—:**

Cangrelor is an intravenous ADP receptor antagonist that leads to potent and reversible inhibition of platelet aggregation. The relative safety and efficacy of some antiplatelet drugs in women has been disputed.

**Methods and Results—:**

The Cangrelor versus Standard Therapy to Achieve Optimal Management of Platelet Inhibition (CHAMPION PHOENIX) trial randomized 11 145 patients undergoing elective or urgent percutaneous coronary intervention to cangrelor or clopidogrel. The primary efficacy end point was the composite of death, myocardial infarction, ischemia-driven revascularization, or stent thrombosis at 48 hours; the key secondary end point was stent thrombosis at 48 hours. The primary safety end point was GUSTO severe bleeding at 48 hours. Of subjects analyzed, 3051 (28%) were female. Cangrelor reduced the odds of the primary end point by 35% in women (adjusted odds ratio [OR], 0.65; 95% confidence interval [CI], 0.48–0.89) and by 14% in men (OR, 0.86; 95% CI, 0.70–1.05; *P* interaction=0.23) compared with clopidogrel. Cangrelor reduced the odds of stent thrombosis by 61% in women (OR, 0.39; 95% CI, 0.20–0.77) and 16% in men (OR, 0.84; 95% CI, 0.53–1.33; *P* interaction=0.11). The odds of severe bleeding were similar in both women and men treated with cangrelor (0.3% versus 0.2%, *P*=0.30 [women]; 0.1% versus 0.1%, *P*=0.41 [men]; *P* interaction=0.88) versus clopidogrel. Cangrelor increased the odds of moderate bleeding in women (0.9% versus 0.3%, *P*=0.02), but not in men (0.2% versus 0.2%, *P*=0.68; *P* interaction=0.040). The net clinical benefit (primary efficacy and safety end point) favored cangrelor in both women (OR, 0.68; 95% CI, 0.50–0.92) and men (OR, 0.87; 95% CI, 0.71–1.06; *P* interaction=0.26).

**Conclusions—:**

In CHAMPION PHOENIX, cangrelor reduced the odds of major adverse cardiovascular events and stent thrombosis in women and men and appeared to offer greater net clinical benefit than clopidogrel.

**Clinical Trial Registration—:**

URL: http://www.clinicaltrials.gov. Unique identifier: NCT01156571.

## Abstract 2

Supplemental Digital Content is available in the text.

Because women remain underrepresented in clinical trials,^1,2^ there exists uncertainty regarding the relative benefit and risk of established and novel antiplatelet therapies in women for the management of cardiovascular disease. In particular, concerns have been raised that the relative risk of thrombosis and bleeding in response to antiplatelet therapy may differ between men and women,^3–6^ and this may therefore influence the net clinical benefit of further intensification of antiplatelet therapy for individual patients.

Clinical Perspective on p 255

Cangrelor is a potent intravenous inhibitor of the P2Y12 receptor that has a rapid onset and a half-life of 3 to 6 minutes, with offset of pharmacodynamic effect within 1 hour.^7^ Cangrelor’s pharmacological profile is consistent regardless of sex, age, or renal or hepatic function.^8,9^ In patients scheduled to undergo elective or urgent percutaneous coronary intervention (PCI), cangrelor reduced the incidence of cardiac ischemic events and stent thrombosis (ST) as compared to clopidogrel without a significant increase in bleeding. Since thrombotic and bleeding complications at the time of PCI remain a major concern, we examined the relative efficacy and safety of cangrelor in women versus men in a prespecified analysis of the CHAMPION (Cangrelor versus Standard Therapy to Achieve Optimal Management of Platelet Inhibition) PHOENIX trial.^10^

## Methods

### Patient Population and Study Treatment

The design and results of the CHAMPION PHOENIX trial have been previously reported.^10,11^ In brief, CHAMPION PHOENIX was a double-blind, double-dummy trial that enrolled 11 145 patients undergoing urgent or elective PCI and randomized them before PCI to either cangrelor (iv bolus then infusion) or clopidogrel (300 mg or 600 mg loading dose) on a background of guideline-recommended therapy. At the end of the infusion, patients were administered either 600 mg of clopidogrel (cangrelor arm) or matching placebo (clopidogrel arm). Aspirin (75 mg to 325 mg) was to be administered in all patients, in addition to a maintenance dose of clopidogrel during the first 48 hours. The choice of periprocedural anticoagulant was left to the discretion of the treating physician. The use of a glycoprotein IIb/IIIa inhibitor was allowed only as rescue therapy during PCI.

Patients were considered eligible for enrollment if they were ≥18 years of age and required PCI for treatment of stable angina or an acute coronary syndrome. Relevant exclusion criteria included administration of a P2Y12 inhibitor or abciximab in the past 7 days or the use of eptifibatide, tirofiban, or fibrinolytic in the 12 hours before randomization. Patients were also excluded if they were perceived to be at increased risk of bleeding, including ischemic stroke in last year or previous hemorrhagic stroke, intracranial aneurysm or arteriovenous malformation, recent (<1 month) trauma or major surgery, those currently receiving warfarin or with signs of active bleeding.

The study protocol was reviewed and approved by institutional review committees and all subjects gave informed consent prior to participation.

### End Points

The primary efficacy end point was the composite of death from any cause, myocardial infarction (MI), ischemia-driven revascularization, or ST at 48 hours after randomization. The key secondary end point was ST at 48 hours which included definite ST according to the Academic Research Consortium definition and intraprocedural ST.^10,12^ As a secondary analysis, efficacy outcomes were collected through 30 days. Safety outcomes were only collected through 48 hours because off-treatment bleeding events that occurred outside of the first 2 days would not be anticipated to be study drug-related based on the rapid pharmacokinetics of cangrelor.

The key safety end point was severe bleeding not related to coronary artery bypass graft according to the GUSTO classification system.^13^ Other bleeding definitions were also applied including the Thrombolysis in Myocardial Infarction (TIMI)^14^ and Acute Catheterization and Urgent Intervention Triage strategY (ACUITY)^15^ bleeding classifications.

All deaths, cardiac ischemic events, and STs were independently adjudicated by a clinical events committee. Bleeding end points were not adjudicated, but were captured by the site.

### Statistical Analysis

Baseline characteristics were compared using t-tests for continuous variables and χ^2^ or Fisher exact tests for categorical variables. Primary efficacy analyses were conducted in the modified intention-to-treat population comprising those patients who underwent PCI and received study drug. Safety analyses were conducted in the safety population that included all patients who underwent randomization and received ≥1 dose of the study drug according to the actual treatment received. End points are reported as incidence rates at 48 hours. Difference between treatment groups was tested using the Pearson χ^2^ statistic as well as odds ratios (ORs) and their corresponding 95% confidence intervals (CIs). Analyses were adjusted for any imbalances (*P*<0.10) in baseline characteristics between randomized treatment arms when stratified by patient sex. Because the female subgroup was relatively underpowered in comparison with the male subgroup, potential confounders identified in the male subgroup were also included as covariates in the female-only models, including previous MI, history of heart failure, peripheral arterial disease, previous coronary artery bypass graft, or abnormal cardiac biomarkers. To assess the homogeneity of the crude odds ratios in the sex subgroup analyses, a Breslow-Day statistic was used. A multivariable model was created to examine independent predictors of the primary efficacy outcome and GUSTO severe or moderate bleeding; a complete list of covariates is included in the online-only Data Supplement. Nominal *P* values are reported, and no adjustment was made for the comparison of multiple outcomes; all tests were 2-sided with a *P* value <0.05 considered to be significant. Analyses were performed using SAS version 9.3 (Cary, NC).

## Results

Of 11 145 patients enrolled in the CHAMPION PHOENIX trial, 3051 subjects (28%) were female. Women were more likely than men to be older and have a history of diabetes mellitus, hypertension, hyperlipidemia, previous stroke or transient ischemic attack, and were more likely to be enrolled in the United States in comparison with other regions (Table 1). Women were more likely than men to be enrolled with a qualifying event of stable angina or non–ST-segment–elevation myocardial infarction whereas men were more frequently enrolled with ST-segment–elevation myocardial infarction. Women tended to have a lower weight, and were less likely to be smokers or have a previous history of MI or coronary revascularization. Women had a lower baseline hemoglobin and hematocrit than men (Table 1). The choice of clopidogrel loading dose and the use of unfractionated heparin and bivalirudin were similar for both sexes, but men were more likely to be administered aspirin. The median duration of PCI was longer in men than women, but the choice of access site (femoral versus radial) was similar for both sexes (Table 1).

**Table 1.**
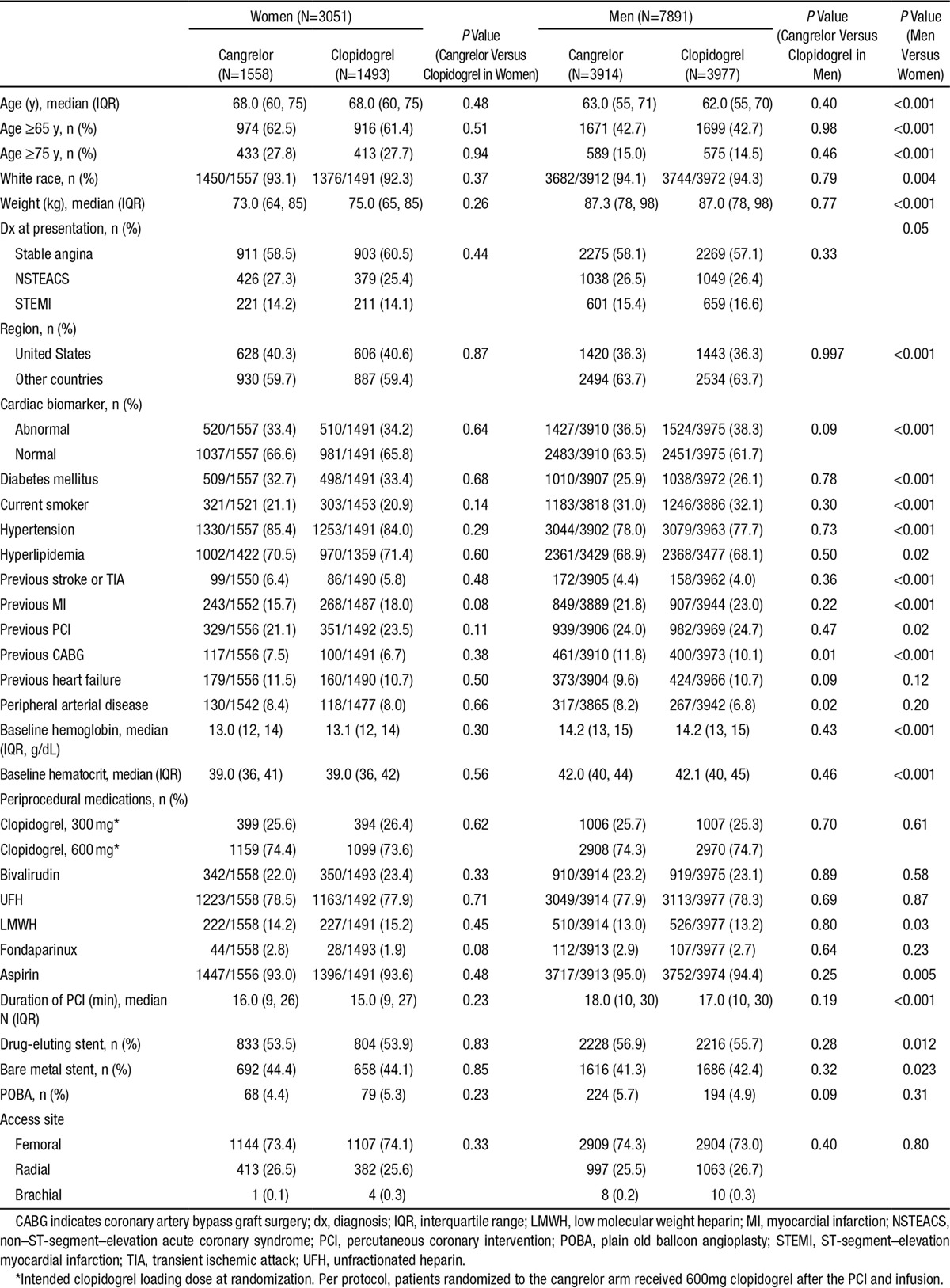
Baseline Characteristics in the CHAMPION PHOENIX Trial Stratified by Patient Sex

After multivariable adjustment, female sex was independently associated with increased odds of the primary efficacy outcome (OR, 1.31; 95% CI, 1.03–1.67) and GUSTO moderate or severe (non–coronary artery bypass graft–related) bleeding (OR, 2.70; 95% CI, 1.19–6.13).

### Efficacy Outcomes With Cangrelor

In women, cangrelor reduced the odds of the primary end point by 35% (adjusted OR, 0.65; 95% CI, 0.48–0.89; *P*=0.01; Figure and Table 2) and reduced the odds of ST by 61% (adjusted OR, 0.39; 95% CI, 0.20–0.77; *P*=0.01) as compared with clopidogrel. In male patients, cangrelor was associated with a 14% reduction in the odds of the primary end point (adjusted OR, 0.86; 95% CI, 0.70–1.05; *P*=0.14; *P* interaction=0.23; Figure and Table 2) and a 16% reduction in the odds of ST (adjusted OR, 0.84; 95% CI, 0.53–1.33; *P*=0.44; *P* interaction=0.11).

**Table 2.**
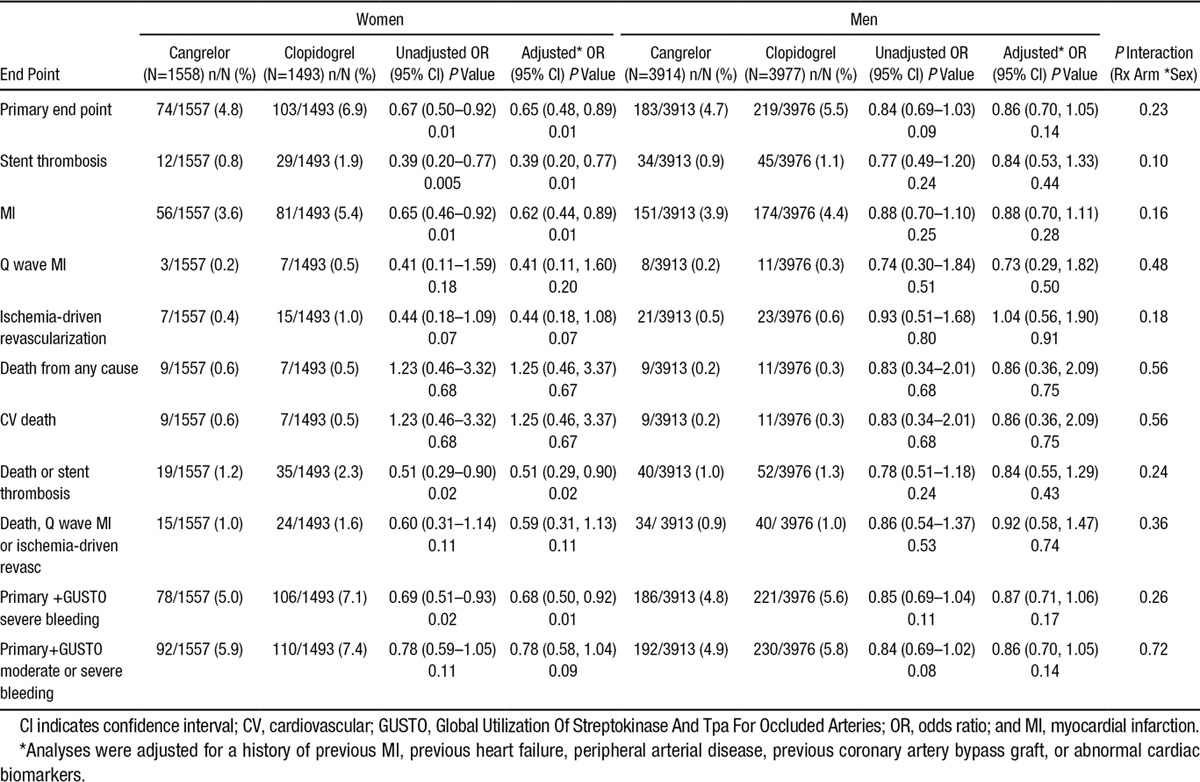
Efficacy and Net Clinical Benefit of Cangrelor Versus Clopidogrel Stratified by Sex at 48 Hours

**Figure.**
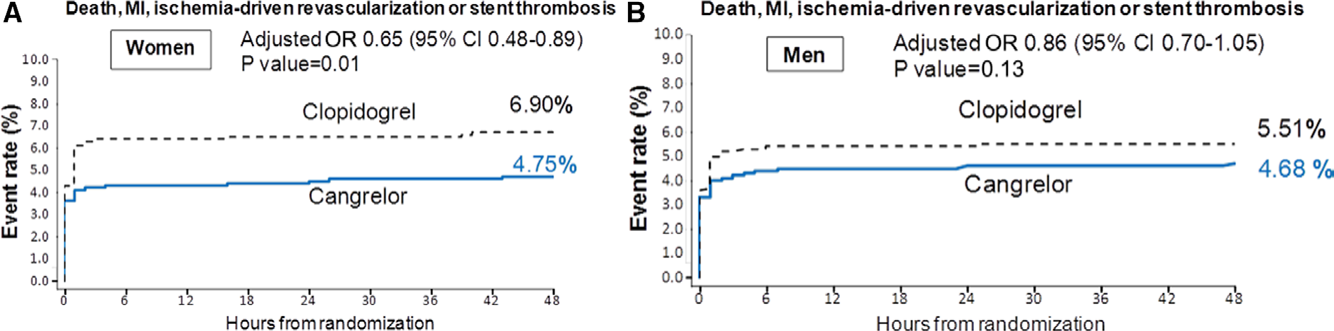
The Kaplan–Meier incidence of the primary end point of death, MI, ischemia-driving revascularization or stent thrombosis at 48 hours in women (**A**) and men (**B**) in the CHAMPION-PHOENIX trial. The interaction between sex and randomized treatment assignment was not significant (*P* interaction=0.23). CI indicates confidence interval; HR, hazard ratio; and MI, myocardial infarction.

The efficacy of cangrelor in women toward reducing the primary end point appeared to be primarily driven by the aforementioned reduction in ST, in addition to a 38% reduction in the odds of MI (adjusted OR, 0.62; 95% CI, 0.44–0.89; *P*=0.01) and a 56% reduction in the odds of ischemia-driven revascularization (adjusted OR, 0.44; 95% CI, 0.18–1.08; *P*=0.07; Table 2). In men, cangrelor was associated with a 12% lower odds of MI (adjusted OR, 0.88; 95% CI, 0.70–1.11; *P*=0.28; *P* interaction=0.15) and a more neutral effect on the odds of ischemia-driven revascularization (adjusted OR, 1.04; 95% CI, 0.56–1.90; *P*=0.91; *P* interaction=0.18; Table 2).

Directionally consistent results were observed when the efficacy of cangrelor by patient sex was examined at 30 days (Table I in the online-only Data Supplement).

### Safety Outcomes With Cangrelor

In both women and men, cangrelor did not increase the odds of the primary safety end point, GUSTO severe or life-threatening bleeding, as compared with clopidogrel (0.3% versus 0.2%, *P*=0.30; 0.1% versus 0.1%, *P*=0.41, respectively; *P* interaction=0.88). However, cangrelor increased the odds of GUSTO moderate bleeding in women when compared with clopidogrel (0.9% versus 0.3%, *P*=0.02; Table 3). In contrast, an excess in GUSTO moderate bleeding was not observed in male patients treated with cangrelor as compared to clopidogrel (0.2% versus 0.2%, *P*=0.68; *P* interaction=0.04). The increase in GUSTO moderate bleeding in women was explained by a higher incidence of blood transfusions in cangrelor-treated women (1.1% versus 0.3%, *P*=0.01), as compared with those treated with clopidogrel. Cangrelor did not increase the odds of blood transfusions in men (0.2% versus 0.3%, *P*=0.56; *P* interaction=0.03). Intracranial hemorrhage was infrequent in both men and women and not significantly increased with use of cangrelor in either sex (Table 3). There were no confirmed fatal bleeding events. Additional bleeding end points are shown in Table 3.

**Table 3.**
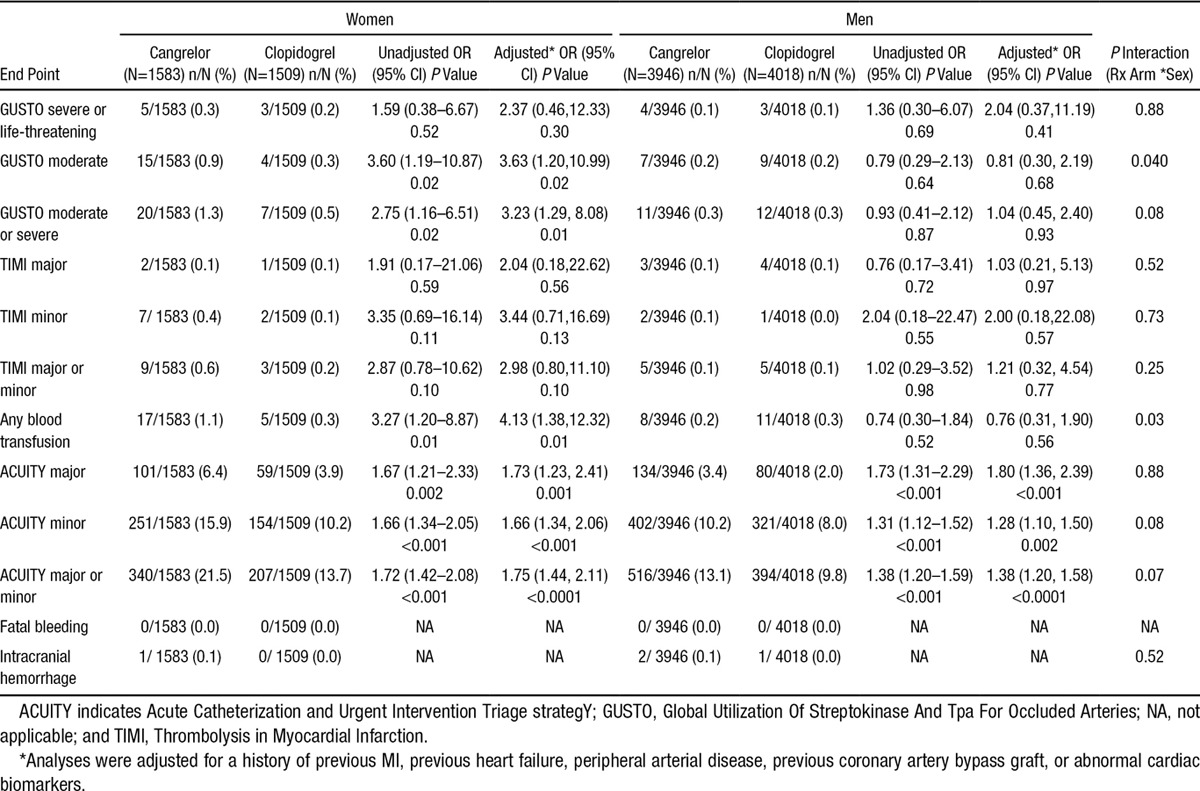
Safety End Points Stratified by Sex in Safety Population

### Net Clinical Benefit

The net clinical benefit (composite of the primary efficacy and safety end points) favored cangrelor as compared with clopidogrel in both women (adjusted OR, 0.68; 95% CI, 0.50–0.92; *P*=0.01) and in men (adjusted OR, 0.87; 95% CI, 0.71–1.06; *P*=0.17; *P* interaction=0.26). Consistent results were observed with cangrelor when moderate bleeding was included in the net benefit outcome (primary end point or GUSTO moderate or severe bleeding) in both women (adjusted OR, 0.78; 95% CI, 0.58–1.04; *P*=0.09) and men (adjusted OR, 0.86; 95% CI, 0.70–1.05; *P*=0.14; *P* interaction=0.73; Table 2).

## Discussion

In the current prespecified subgroup analysis of the CHAMPION PHOENIX trial, cangrelor demonstrated a consistent reduction in the odds of major adverse cardiovascular events (MACE) and ST in both male and female patients. In particular, cangrelor reduced the odds of the primary end point by 32% and the odds of ST by 61% in female subjects as compared with those treated with clopidogrel. By comparison, cangrelor was associated with a 15% reduction in the odds of MACE and a 16% reduction in the odds of ST in male subjects without evidence of heterogeneity by sex. Although some previous reports have demonstrated sex-based differences in response to some antiplatelet therapies,^3–5,16^ the observed benefit with cangrelor was consistent in female and male patients.

Although it remains unclear whether true biological differences exist at the level of the platelet, several previous studies have demonstrated that women have higher baseline platelet reactivity as compared with men.^17–19^ Therefore, a strategy of platelet inhibition for the prevention of cardiovascular events could offer particular appeal in women. Although aspirin has a comparable pharmacodynamic response in both women and men, women have higher on-treatment platelet reactivity on aspirin owing to their higher platelet reactivity levels at baseline.^18^ For clopidogrel, some reports have suggested that its pharmacodynamic response in women may be attenuated thereby leading to a higher proportion of clopidogrel hyporesponders.^20,21^ These observations have contributed to a greater need to adequately evaluate novel antiplatelet therapies in both women and men. In a meta-analysis of trials for aspirin use in the primary prevention of cardiovascular disease, aspirin reduced cardiovascular events in both women and men, but the benefit of aspirin in women was primarily toward stroke reduction, whereas aspirin reduced the risk of MI in men.^5^ In the Antithrombotic Trialists’ Collaboration, the clinical benefit of aspirin in women was diminished when compared with men when primary and secondary prevention studies were combined, but this difference was no longer significant once adjusted for multiple comparisons.^22^ Subsequently, in a meta-analysis of randomized trials that studied clopidogrel, clopidogrel had comparable efficacy toward MACE reduction in both women and men, but the benefit of clopidogrel in women was driven primarily by a reduction in MI, whereas men had a more robust reduction in the risk of MI, stroke, and all-cause mortality.^23^ A meta-analysis of trials of glycoprotein IIb/IIIa inhibitors uncovered a significant interaction in efficacy between men and women with a potential signal toward harm in female patients. However, when the female population was restricted to those with elevated troponin levels, there appeared to be a more consistent benefit regardless of sex.^3^ Notably in the current analysis, cangrelor demonstrated marked efficacy in women during the periprocedural period even though the majority of patients were enrolled with stable angina and had normal baseline cardiac biomarkers.

In the current analysis, although severe and life-threatening bleeds were similar regardless of treatment arm, we observed an increase in the risk of GUSTO moderate bleeding with cangrelor in women (0.9% versus 0.3%) that was not seen in male patients (0.2% versus 0.2%; *P* interaction=0.04). However, it is relevant that the definition of a GUSTO moderate bleeding event includes any bleeding that requires transfusion (without hemodynamic compromise). Because the decision to transfuse is often based on the patient’s hemoglobin concentration, it is notable that women had a lower baseline hemoglobin concentration than men at study entry. Therefore, it is plausible that this baseline difference between sexes may have contributed to a higher frequency of blood transfusions in female patients. To that end, the odds of bleeding with cangrelor were more comparable in both women and men when alternate bleeding definitions were applied. Nonetheless, women overall had a higher incidence of bleeding than men regardless of therapy, as has been noted in prior PCI analyses,^24^ and a nonsignificant trend was observed across alternate bleeding definitions toward more bleeding in cangrelor-treated women than those treated with clopidogrel. One cannot exclude that higher age, a lower body mass index, and other comorbidities in women may have explained the observed bleeding signal. Importantly, the net clinical benefit that considered both efficacy and bleeding (including GUSTO severe or moderate bleeding events) continued to favor cangrelor in both women and men because of the marked reduction in MACE in female patients treated with cangrelor.

Limitations of the current analysis include the fact that tests for heterogeneity are conservative and the trial was not powered to examine outcomes within individual subgroups. Exploration within patient subgroups may increase the risk of a false-positive finding and should therefore be considered exploratory. As well, randomization was not stratified on the basis of patient sex and therefore we cannot exclude that unmeasured confounders may have differed across randomized treatment arms in either women or men. However, very few imbalances were observed between randomized treatment arms when stratified by sex because of the large size of the trial and all analyses were adjusted for potential confounders. Although the GUSTO moderate bleeding definition is based on criteria that may have been influenced by the patient’s baseline hemoglobin, multiple bleeding definitions were included in the current analysis.

Because CHAMPION PHOENIX enrolled patients with either stable angina (58.1% of patients enrolled) or acute coronary syndrome, the trial did not compare the efficacy or safety of cangrelor to prasugrel or ticagrelor, which are now endorsed preferentially over clopidogrel in appropriate patients in the setting of acute coronary syndrome.^25–27^ These therapies are not currently recommended in the setting of elective PCI and were therefore not studied in the current trial. The current substudy is underpowered to examine whether efficacy and safety were comparable in women or men within the acute coronary syndrome or stable angina subgroups.

In summary, the current findings provide important reassurance that the efficacy and net clinical benefit of cangrelor at the time of PCI is maintained in female patients, a population historically understudied in cardiology trials. Efforts should continue to identify patient characteristics that may help to find those individuals who will derive the greatest net clinical benefit from novel antiplatelet therapies.

## Acknowledgments

We thank Steven E. Elkin, MS and Debra Bernstein, PhD of The Medicines Company for their statistical support, along with Yuyin Liu, MS and Lanyu Lei, MS of the Harvard Clinical Research Institute for their independent verification of the analyses. Harvard Clinical Research Institute received funding from The Medicines Company for these analyses.

## Sources of Funding

The CHAMPION PHOENIX trial was funded by The Medicines Company.

## Disclosures

Dr O’Donoghue has received research grants from Merck, GlaxoSmithKline, Eisai, and AstraZeneca. Dr White has received research grants from Sanofi, Eli Lilly, The Medicines Company, National Institutes of Health, Pfizer, Roche, Johnson & Johnson, Schering Plow, Merck Sharpe & Dohme AstraZeneca, GlaxoSmithKline, Daiichi Sankyo, Pharma Development, and Bristol-Myers Squibb and has served on the Advisory Boards of Merck Sharpe & Dohme, Roche, and Regado Biosciences. Dr Gibson has received funding from or has been a consultant for Angel Medical Corporation, AstraZeneca, Atrium Medical Systems, Baxter Healthcare, Bayer, Cardiovascular Research Foundation, Consensus Medical Communications, CSL Behring, Cytori Therapeutics, Daiichi Sankyo, Eli Lilly, Exeter Group, Genentech, GlaxoSmithKline, Ikaria, Janssen Pharmaceuticals, Johnson & Johnson, Lantheus Medical Imaging, Merck, Ortho-McNeil, Portola Pharmaceuticals, Roche Diagnostics, Sanofi, StealthPeptides, St. Jude Medical, The Medicines Company, UpToDate in Cardiovascular Medicine Volcano Corp, and Walk Vascular. Dr Hamm has received honoraria from Abbott, AstraZeneca, Bayer, Berlin Chemie, Boehringer Ingelheim, Merck Sharpe & Dohme, Bristol-Myers Squibb, BRAHMS, Daiichi Sankyo, Essex, GlaxoSmithKline, Medtronic, Lilly, Sanofi, Correvio, Pfizer, Roche, The Medicines Company, Boston Scientific, and Gilead. Dr Mahaffey’s financial disclosures prior to August 1, 2013, can be viewed at https://www.dcri.org/about-us/conflict-of-interest/Mahaffey-COI_2011-2013.pdf; disclosures after August 1, 2013, can be viewed at http://med.stanford.edu/profiles/kenneth-mahaffey. Dr Price has received consulting honoraria from AstraZeneca, Merck, Terumo, Spectranetics, The Medicines Company, Medtronic, St. Jude Medical, and Boston Scientific and speaker honoraria from AstraZeneca. Dr Steg has received personal fees from Amarin, AstraZeneca, Bayer, Boehringer Ingelheim, Bristol-Myers Squibb, Daiichi Sankyo, GlaxoSmithKline, Lilly, Merck Sharpe & Dohme, Novartis, Otsuka, Pfizer, Roche, Sanofi, Servier, The Medicines Company, and Vivus. Dr Stone has been a consultant for Abbott Vascular, Boston Scientific, Bristol-Myers Squibb–Sanofi partnership, Eli Lilly, Daiichi Sankyo, AstraZeneca, and The Medicines Company. Dr Harrington has received research grants from AstraZeneca, Bristol-Myers Squibb, Sanofi, The Medicines Company, Eli Lilly, Daiichi Sankyo, GlaxoSmithKline, Johnson & Johnson, Portola, Merck, and Regado and has been a consultant for Sanofi, Bristol-Myers Squibb, Merck, Johnson & Johnson, and Gilead. Drs Prats, Deliargyris, and Liu are employees of The Medicines Company. Dr Deepak L. Bhatt discloses the following relationships - Advisory Board: Cardax, Elsevier Practice Update Cardiology, Medscape Cardiology, Regado Biosciences; Board of Directors: Boston VA Research Institute, Society of Cardiovascular Patient Care; Chair: American Heart Association Get With The Guidelines Steering Committee; Data Monitoring Committees: Duke Clinical Research Institute, Harvard Clinical Research Institute, Mayo Clinic, Population Health Research Institute; Honoraria: American College of Cardiology (Senior Associate Editor, Clinical Trials and News, ACC.org), Belvoir Publications (Editor in Chief, Harvard Heart Letter), Duke Clinical Research Institute (clinical trial steering committees), Harvard Clinical Research Institute (clinical trial steering committee), HMP Communications (Editor in Chief, *Journal of Invasive Cardiology*), *Journal of the American College of Cardiology* (Guest Editor; Associate Editor), Population Health Research Institute (clinical trial steering committee), Slack Publications (Chief Medical Editor, Cardiology Today’s Intervention), WebMD (CME steering committees); Other: Clinical Cardiology (Deputy Editor); Research Funding: Amarin, AstraZeneca, Bristol-Myers Squibb, Eisai, Ethicon, Forest Laboratories, Ischemix, Medtronic, Pfizer, Roche, Sanofi Aventis, The Medicines Company (including for his role as Co-Chair of CHAMPION PHOENIX); Site Co-Investigator: Biotronik, St. Jude Medical; Trustee: American College of Cardiology; Unfunded Research: FlowCo, PLx Pharma, Takeda. The other authors report no conflicts.

## Supplementary Material

## CLINICAL PERSPECTIVE

Cangrelor is an intravenous ADP receptor antagonist that leads to potent and reversible inhibition of platelet aggregation. The relative safety and efficacy of some antiplatelet drugs in women has been disputed. The CHAMPION PHOENIX trial randomized 11 145 patients undergoing elective or urgent percutaneous coronary intervention to cangrelor or clopidogrel. Overall the efficacy of cangrelor was similar in men and women with an observed trend toward greater benefit in women. The odds of severe bleeding with cangrelor were similar in men and women; however, cangrelor increased the odds of moderate bleeding in women but not in men (*P* interaction=0.045). In part, this might be explained by lower baseline hemoglobin concentration thereby contributing to a higher frequency of blood transfusions in women. Overall, the net clinical benefit favored cangrelor over clopidogrel in both women and men undergoing percutaneous coronary intervention.
