# Supplementary material for: Efficacy and Safety of Cangrelor in Women Versus Men During Percutaneous Coronary Intervention: Insights From the Cangrelor versus Standard Therapy to Achieve Optimal Management of Platelet Inhibition (CHAMPION PHOENIX) Trial
Source: Circulation. 2016 Jan 18;133(3):248–55. doi: 10.1161/CIRCULATIONAHA.115.017300 (PMC4894784; doi:10.1161/CIRCULATIONAHA.115.017300)
Supplement: Supplementary file 1 [file cir-133-248-s001.pdf]

Supplemental Material

Supplementary methods

A multivariable model was created to examine independent predictors of the primary efficacy outcome and GUSTO severe or moderate bleeding and included the following covariates: sex, age ≥75 years, white race, smoker (yes vs no), weight (<60kg), prior MI, peripheral arterial disease, congestive heart failure, diabetes mellitus, prior cerebrovascular event, prior PCI, prior CABG, received drug eluting stent (yes vs no), United States (yes vs no), timing of study drug administration (before or after start of PCI), loading dose (300 or 600mg), baseline aspirin dose (low vs high), anticoagulant use (bivalirudin only vs heparin only), GPI used (yes vs no), vessels with stenosis >50% (≥2), qualifying event (stable angina vs ACS), biomarker positive (yes vs no), infusion duration (≤129 minutes), PCI duration, total stents placed, total stent length and randomized treatment arm.

Supplementary Table 1: Efficacy of cangrelor versus clopidogrel stratified by sex at 30 days.

	**Women**				**Men**				**P interaction (Rx arm *sex)**
	**Cangrelor (N=1558)**	**Clopi (N=1493)**	**Unadjusted OR (95% CI) P value**	**Adjusted* OR (95% CI) P value**	**Cangrelor (N=3914)**	**Clopi (N=3977)**	**Unadjusted OR (95% CI) P value**	**Adjusted* OR (95% CI) P value**	
Death, MI, ischemia-driven revascularization or stent thrombosis	100/ 1555 (6.4)	123/ 1490 (8.3)	0.76 (0.58, 1.00) 0.053	0.75 (0.57, 0.99) 0.04	226/ 3907 (5.8)	257/ 3967 (6.5)	0.89 (0.74, 1.07) 0.20	0.90 (0.75, 1.09) 0.27	0.38
Stent thrombosis	17/ 1555 (1.1)	35/ 1490 (2.3)	0.46 (0.26, 0.82) 0.008	0.46 (0.25, 0.82) 0.009	54/ 3907 (1.4)	69/ 3967 (1.7)	0.79 (0.55, 1.13) 0.20	0.86 (0.60, 1.24) 0.42	0.12
Death	26/ 1555 (1.7)	22/ 1490 (1.5)	1.13 (0.64, 2.01) 0.67	1.16 (0.64, 2.08) 0.63	34/ 3907 (0.9)	33/ 3967 (0.8)	1.05 (0.65, 1.69) 0.85	1.11 (0.68, 1.80) 0.68	0.83
CV Death	23/ 1555 (1.5)	18/ 1490 (1.2)	1.23 (0.66, 2.28) 0.52	1.19 (0.63, 2.23) 0.60	25/ 3907 (0.6)	28/ 3967 (0.7)	0.91 (0.53, 1.56) 0.72	0.97 (0.56, 1.68) 0.92	0.47
Myocardial infarction	61/ 1555 (3.9)	85/ 1490 (5.7)	0.67 (0.48, 0.95) 0.02	0.65 (0.46, 0.91) 0.01	164/ 3907 (4.2)	187/ 3967 (4.7)	0.89 (0.71, 1.10) 0.27	0.89 (0.71, 1.10) 0.28	0.18
Q wave myocardial infarction	4/ 1555 (0.3)	7/ 1490 (0.5)	0.55 (0.16, 1.87) 0.33	0.55 (0.16, 1.89) 0.34	10/ 3907 (0.3)	15/ 3967 (0.4)	0.68 (0.30, 1.51) 0.34	0.67 (0.30, 1.50)0.33	0.78
Ischemia-driven revascularization	20/ 1555 (1.3)	21/ 1490 (1.4)	0.91 (0.49, 1.69) 0.77	0.92 (0.49, 1.70) 0.78	36/ 3907 (0.9)	45/ 3967 (1.1)	0.81 (0.52, 1.26) 0.35	0.85 (0.54, 1.34) 0.49	0.76
Death, Q wave MI or ischemia-driven revasc	44/ 1555 (2.8)	43/ 1490 (2.9)	0.98 (0.64, 1.50) 0.58	0.99 (0.64, 1.53) 0.97	73/ 3907 (1.9)	81/ 3967 (2.0)	0.91 (0.66, 1.26) 0.58	0.95 (0.69, 1.32) 0.77	0.80
Death or stent thrombosis	38/ 1555 (2.4)	48/ 1490 (3.2)	0.75 (0.49, 1.16) 0.20	0.75 (0.48, 1.16) 0.19	72/ 3907 (1.8)	86/ 3967 (2.2)	0.85 (0.62, 1.16) 0.31	0.91 (0.66, 1.25) 0.56	0.66

* Analyses were adjusted for a history of prior MI, prior heart failure, peripheral arterial disease, prior CABG or abnormal cardiac biomarkers.
